# *miR-16* and *miR-21* Expression in the Placenta Is Associated with Fetal Growth

**DOI:** 10.1371/journal.pone.0021210

**Published:** 2011-06-15

**Authors:** Matthew A. Maccani, James F. Padbury, Carmen J. Marsit

**Affiliations:** 1 Department of Pathology and Laboratory Medicine, Brown University, Providence, Rhode Island, United States of America; 2 Department of Pediatrics, Women and Infants Hospital, Providence, Rhode Island, United States of America; 3 Center for Environmental Health and Technology, Department of Community Health, Brown University, Providence, Rhode Island, United States of America; Tulane University Health Sciences Center, United States of America

## Abstract

**Background:**

Novel research has suggested that altered miRNA expression in the placenta is associated with adverse pregnancy outcomes and with potentially harmful xenobiotic exposures. We hypothesized that aberrant expression of miRNA in the placenta is associated with fetal growth, a measurable phenotype resulting from a number of intrauterine factors, and one which is significantly predictive of later life outcomes.

**Methodology/Principal Findings:**

We analyzed 107 primary, term, human placentas for expression of 6 miRNA reported to be expressed in the placenta and to regulate cell growth and development pathways: *miR-16*, *miR-21*, *miR-93*, *miR-135b*, *miR-146a*, and *miR-182*. The expression of *miR-16* and *miR-21* was markedly reduced in infants with the lowest birthweights (p<0.05). Logistic regression models suggested that low expression of *miR-16* in the placenta predicts an over 4-fold increased odds of small for gestational age (SGA) status (p = 0.009, 95% CI = 1.42, 12.05). Moreover, having both low *miR-16* and low *miR-21* expression in the placenta predicts a greater increase in odds for SGA than having just low *miR-16* or *miR-21* expression (p<0.02), suggesting an additive effect of both of these miRNA.

**Conclusions/Significance:**

Our study is one of the first to investigate placental miRNA expression profiles associated with birthweight and SGA status. Future research on miRNA whose expression is associated with *in utero* exposures and markers of fetal growth is essential for better understanding the epigenetic mechanisms underlying the developmental origins of health and disease.

## Introduction

Fetal development represents a critical period during which perturbations to the intrauterine environment through various factors in the extrauterine environment can have major ramifications on not only the proper growth and development of the fetus but also on risk for disease later in life [1]. Barker and Hales [1] hypothesized that fetuses receive a poor or rich maternal forecast depending, in part, on the intrauterine and extrauterine conditions during pregnancy, but that this maternal forecast may not always accurately predict the post-birth environment and that such mismatches give rise to disease risk later in life. This has played out in a number of epidemiologic studies linking low birthweight with morbidity and mortality in early infancy [2] as well as with an increased risk for certain diseases later in life, particularly coronary heart disease, diabetes mellitus type 2, and hypercholesterolemia [2].

The placenta is of critical importance to ensure the proper growth and development of the fetus while *in utero*. It is involved in providing the fetus with nutrients and is involved in waste and gas exchange. The placenta's metabolic activity is crucial for protecting the fetus from potentially harmful maternal factors and xenobiotic toxicants that may alter fetal growth and development. The environment during pregnancy is thought to impact the appropriate function of the placenta during development, thus identification of alterations to the placenta and to placental gene expression may serve as a record of *in utero* exposures and of the intrauterine and extrauterine environments during pregnancy [3].

The mechanisms by which in utero exposures may dysregulate the regulatory mechanisms of the placenta continue to be studied. One mode of alteration may be through the aberrant expression of microRNA (miRNA), 21–25 nucloeotide long non-coding RNA involved in post-transcriptional gene regulation [4], [5]. miRNA base-pair to the 3′-untranslated region of target mRNA and effectively silence gene expression by a mechanism of either translational repression or direct mRNA degradation. The particular mechanism of this post-transcriptional regulation depends greatly on the degree of complementarity of the miRNA to its mRNA target. Previous work has shown that partial complementarity of a miRNA to an mRNA target may result in effective repression of translation; therefore, a single miRNA can regulate a vast number of genes [5]. Through this mechanism of post-transcriptional gene regulation, miRNA have been shown to regulate a number of key cellular functions including migration, invasion, growth, and death [6]. miRNA exhibit tissue-specific expression and function and have been shown to be expressed in the placenta in addition to a variety of other tissues [7]. Alterations to placental miRNA expression have been associated with *in utero* exposures [8], [9] and adverse pregnancy outcomes [10], [11], [12], [13].

Since miRNA have been described as playing important roles in development and are susceptible to the environment, we sought to further characterize the expression of six candidate miRNA previously shown to be expressed in the placenta and previously reported to target genes in pathways crucial for regulating key cell processes – *miR-16* [9], [14], *miR*-2*1* [9], [15], *miR-93* [12], [13], *miR-135b* [11], *miR-146a* [9], [16], and *miR-182* [10] – in a large series of human placentas for associations with fetal growth.

## Results

**Figure 1:**
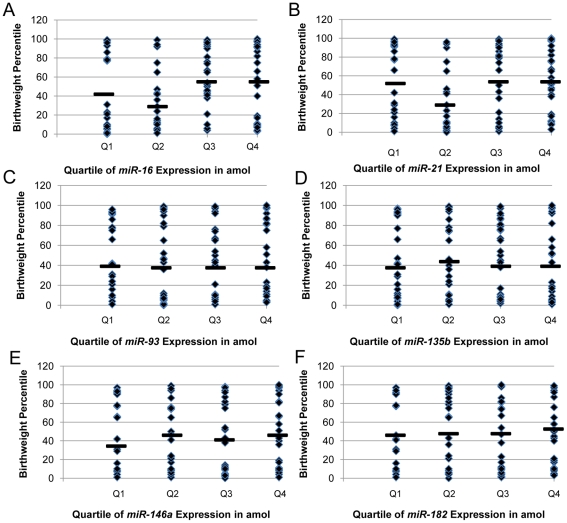
Distribution of infant birthweights (y-axis) by primary term human placenta miRNA expression quartiles (x-axis). (A) *miR-16* (p = 0.04), (B) *miR-21* (p = 0.02), (C) *miR-93* (p = 0.88), (D) *miR-135b* (p = 0.84), (E) *miR-146a* (p = 0.46), and (F) *miR-182* (p = 0.55). Black bars indicate median of birthweight percentile within each quartile. Kruskal-Wallis tests revealed that birthweight percentile significantly differed across quartiles of *miR-16* and *miR-21* expression (p<0.05).

One hundred seven human placenta samples were analyzed for the expression of candidate miRNA previously shown to be expressed in the placenta and involved in regulating cell growth and developmental processes by targeting genes in a variety of cell growth and cell functioning pathways, specifically, *miR*-16, *miR-21*, *miR-93*, *miR-135b*, *miR-146a*, and *miR-182*. The demographics of the sample population are given in **Table 1** **.** Of note, the study population was oversampled for small for gestational age (SGA) infants, defined as infants whose birth weight was the lowest 10^th^ percentile for their gestational age, calculated as in Fenton [17]. Placentas from SGA neonates comprised approximately 30% of the study population. **Table 2** describes the expression of the 6 candidate miRNA in all of the 107 samples, based on qRT-PCR and absolute quantification from a standard curve. The range of expression values differed for the miRNAs, with *miR-146a* and *miR-182* exhibiting expression in the 0.001–3.95 amol range, while *miR-16* and miR-*21* expression was 2 orders of magnitude greater with expression ranging from 3.53–434.74 amol range. Within each miRNA, the majority of the samples exhibited relatively homogenous low levels of expression, but the distributions of expression exhibited a right skew, suggesting there were still a number of samples with relatively moderate to high expression. As these data were not normally distributed, and as we hypothesized that aberrant high or low expression may be associated with exposures or outcomes, the expression profiles were split into quartiles, and birthweight percentile was plotted by quartile of miRNA expression (**Figure 1**). Such an analytical strategy using birthweight percentile as a continuous variable allowed us to gain a broader observation of association of birthweight percentile with miRNA expression. Kruskal-Wallis tests were then used to assess differences in birthweight percentile across quartiles of miRNA expression (**Figure 1**). Analysis revealed that birthweight percentile significantly differed across quartiles of *miR-16* and *miR-21* expression, p = 0.04 and p = 0.02, respectively.

**Table 1:** Demographics of the study population (n = 107).

	SGA	Non-SGA	p
**Birthweight, grams, mean (sd)**	2501 (326)	3652 (585)	<0.0001
**Gestational age, weeks, mean (sd)**	38.7 (1.35)	39.0 (1.07)	0.3
**Birthweight by gestational age status, n (%)**	32 (30%)	75 (70%)	n/a
**Infant gender, n (%)**			0.03
**Female**	23 (72%)	37 (49%)	
**Male**	9 (28%)	38 (51%)	
**Mode of delivery** * **, n (%)**			0.53
**Caesarian section**	10 (31%)	29 (39%)	
**Vaginal delivery**	21 (66%)	46 (61%)	
**Maternal age, years, mean (sd)**	28.06 (6.63)	30.04 (5.63)	0.15
**Maternal ethnicity** ** **, n (% )**			0.76
**Non-white**	8 (25%)	16 (21%)	
**White**	24 (75%)	56 (75%)	
**Maternal cigarette smoking during pregnancy** ***			0.01
**No**	28 (88%)	72 (96%)	
**Yes**	4 (12%)	1 (1%)	
**Relative weight gained during pregnancy, % of prepregnancy weight, mean (sd)** ****	19.9 (0.09)	21 (0.11)	0.6
**Maternal insurance**			0.36
**Public**	15 (47%)	29 (39%)	
**Private**	17 (53%)	46 (61%)	

*One sample was missing mode of delivery data.

**Three samples were missing maternal ethnicity data.

***Two samples were missing maternal cigarette smoking during pregnancy data.

****Two samples were missing weight gained data.

Note: Tests for the difference in specific clinical or demographic factors between the 2 groups (SGA and non-SGA). T-test was used to examine differences in continuous variables, and χ2-tests for categorical variables.

**Table 2:** Expression of *miR-16*, *miR-21*, *miR-93*, *miR-135b*, *miR-146a*, and *miR-182* determined through qRT-PCR in 107 primary human term placenta samples.

	Median (amol)	Range (amol)
*miR-16*	18.64	3.53–399.50
*miR-21*	54.86	5.25–434.74
*miR-93*	4.26	0.14–118.39
*miR-135b*	4.72	0.13–216.92
*miR-146a*	0.1	0.002–3.95
*miR-182*	0.23	0.001–3.63

Observing that expression in the lowest quartiles of *miR-16* and *miR-21* was associated with reduced birthweight percentile, we more specifically examined the association between low expression (≤median vs. >median) of the miRNA and infants considered small for gestational age (SGA), using logistic regression to control for potential confounders. SGA status served as a more clinically-relevant marker of the multitude of *in utero* conditions which may comprise an adverse intrauterine environment which may be ultimately associated with low birthweight. These models (**Table 3**) demonstrate that low *miR-16* expression in the placenta predicts an odds of 4.13 for SGA (95% CI = 1.42, 12.05) compared to infants with high placenta *miR-16* expression, controlled for confounders. Additionally, low *miR-21* expression in the placenta predicts an intriguing but nonetheless statistically insignificant, 2.43-fold increased odds for SGA (95% CI = 0.93, 6.37) compared to infants with high placenta *miR-21* expression controlled for confounders. Maternal cigarette smoking during pregnancy, as expected, also demonstrated greatly increased risks for growth restriction in both models.

**Table 3:** Logistic regression for the association between individual miRNA expression and SGA status.

Effect	Odds Ratio	95% Wald Confidence Limits	p
*miR-16* Expression in Placenta, n (%)			
High n = 52 (51%)	Reference		
Low n = 50 (49%)	4.13	1.42–12.05	0.009
Maternal Smoking During Pregnancy, n (%)			
No n = 97 (95%)	Reference		
Yes n = 5 (5%)	22.18	1.72–286.86	0.018

Effect	Odds Ratio	95% Wald Confidence Limits	p
*miR-21* Expression in Placenta, n (%)			
High n = 52 (51%)	Reference		
Low n = 50 (49%)	2.43	0.93–6.37	0.069
Maternal Smoking During Pregnancy, n (%)			
No n = 97 (95%)	Reference		
Yes n = 5 (5%)	10.82	0.82–143.58	0.071

Also included in models: Relative Weight Gained During Pregnancy, Maternal Ethnicity, Maternal Age, Delivery Method, Insurance, and Infant Gender.

Samples lacking one or more piece of covariate data were excluded from the model.

As both miRNA demonstrated independent correlations with SGA status, we further examined if there was an interaction between expression of these 2 miRNA in their association with infant growth outcome. This model (**Table 4**) demonstrated that compared to infants having high expression (>median) of both miRNA, infants with low *miR-21* only or low *miR-16* only had non-significant elevation in SGA risk, but infants exhibiting reduced expression of both *miR-16* and *miR-21* were significantly more likely to be classified as SGA (OR 5.38, 95% CI 1.52, 19.01). Although a likelihood ratio test suggested no significant multiplicative interaction between *miR-16* and *miR-21* (p>0.05), there was a significant trend for increased risk of being classified as SGA from having only *miR-21* reduced or only *miR-16* reduced in expression to having both reduced in expression (p<0.02).

**Table 4:** Logistic regression to examine the interaction of low *miR-16* and low *miR-21* expression on the association with SGA status.

Effect	Odds Ratio	95% Wald Confidence Limits
*miR-21* and *miR-16*, n (%)		
Both High, n = 36 (35%)	Reference	
Low *miR-21* only, n = 16 (16%)	1.54	0.29–8.21
Low *miR-16* only, n = 16 (16%)	3.35	0.69–16.32
Both Low, n = 34 (33%)	5.38	1.52–19.01

Also included in model: Relative Weight Gained During Pregnancy, Maternal Ethnicity, Maternal Age, Delivery Method, Insurance, and Infant Gender.

p for trend (p<0.02).

Samples lacking one or more piece of covariate data were excluded from the model.

**Figure 2:**
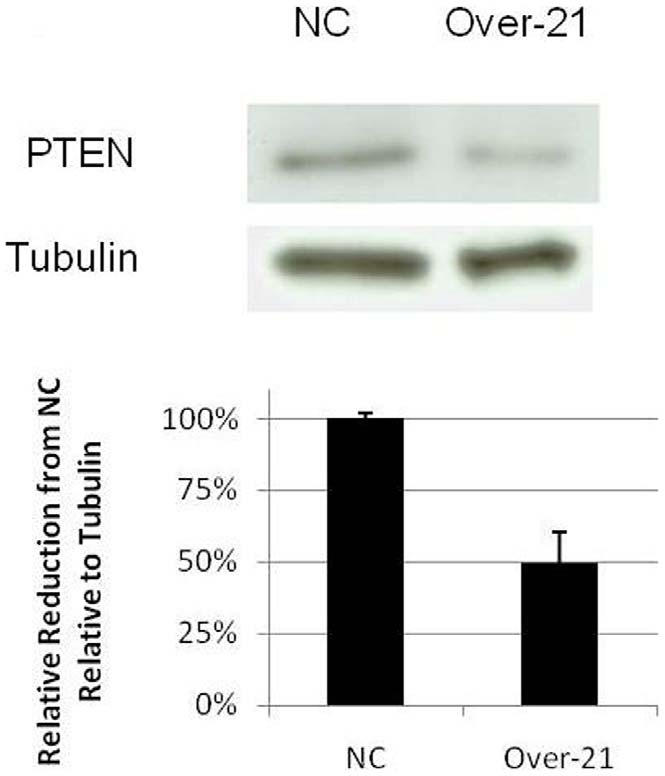
TCL-1 cells overexpressing *miR-21* express approximately 50% less PTEN protein than TCL-1 cells transfected with negative control (p<0.05).

Previous work has empirically validated PTEN as a target of *miR-21* in a variety of cancers [18], [19]. We overexpressed *miR-21* in TCL-1 placental cells, a third trimester human placental cell line, and used Western blot to assess PTEN protein levels in cells overexpressing *miR-21* versus cells transfected with negative control. Cells overexpressing *miR-21* had approximately 50% less PTEN protein relative to tubulin than cells transfected with negative control (**Figure 2**), suggesting that PTEN is a target of *miR-21* in TCL-1 cells.

## Discussion

We have demonstrated the expression of key candidate miRNA in a large, population-based series of primary human placenta samples and their association with poor fetal growth, specifically identifying that reduced expression of *miR-16* and *miR-21* are significantly associated with growth restriction. A number of groups have previously described placental miRNA expression associations with maternal conditions such as preeclampsia [10], with maternal cigarette smoking during pregnancy [20], and as markers of pregnancy itself [11]. Furthermore, more is being uncovered about the role of *miR-16* and *miR-21* in regulating key cellular processes, especially the involvement of *miR-16* in regulating cell cycle progression [14] and *miR-21*'s capability of regulating cell cycling and cell proliferation [15].

Differences in birthweight percentile were examined across quartiles of miRNA expression (**Figure 1**). Birthweight percentile significantly differed across quartiles of *miR-16* and *miR-21* expression, p = 0.04 and p = 0.02, respectively. Further analysis revealed that Q2, the moderate-low expression of *miR-16* and *miR-21*, was especially associated with lower birthweight percentile; this is an intriguing observation possibly suggesting that the moderate-low expression of *miR-16* and *miR-21* associated with lower birthweight percentiles more than the extreme low expression of *miR-16* and *miR-21*. These data suggest that lower expression of *miR-16* and *miR-21* in placenta does associate with lower birthweight percentiles but that the moderate-low expression of these miRNA in placenta may be particularly associative with reduced birthweight; such an observation merits further investigation in the future.

*miR-21* has been described as an oncogene, plays a role in enhancing tumor phenotypes including proliferation and migration, and has been shown to target a number of key regulators of these processes, including but not limited to PLAG1 [21] and PTEN [18], [19]. Meng and coworkers showed that *miR-21* regulates PTEN in human hepatocellular cancer [19], and Lou and colleagues demonstrated that in ovarian epithelial carcinomas, *miR-21* promotes proliferation, invasion and migration abilities by inhibiting PTEN [18]. Previous work has demonstrated that PTEN is expressed in the placenta under normal conditions and may have expression profiles which differ associated with pregnancy stage [22]. Our proof-of-principle data suggest that TCL-1 placental cells overexpressing *miR-21* have approximately 50% less PTEN protein than cells transfected with negative control, suggesting that PTEN may be a target of *miR-21* in TCL-1 cells. As the function of the placenta, though, is to promote fetal growth through its own proliferation and invasion into the maternal decidua, downregulation of *miR-21* in the placenta could, through dysregulation of PTEN, result in decreased invasion of the maternal decidua, decreased migration, and decreased growth – the opposite of what has been observed to occur in the case of upregulated *miR-21* [19].

As with many miRNA, *miR-16* exhibits tissue-specific function and expression. In a number of cancer cell lines, *miR-16* has been shown to be involved in the induction of apoptosis by targeting *BCL-2* [23] and in cell cycle regulation by targeting CDK6 [24], CDC27 [25], and CARD10 [26]. In other cell types, *miR-16* has different functions, such as targeting HMGA1 and Caprin-1 [27], further suggesting that *miR-16* may have cell-type function and expression [23], [27]. Dysregulation of *miR-16* in the placenta may lead to aberrant expression of its targets and may lead to functional and developmental abnormalities in the placenta that might result in reduced infant birthweight. Mechanistic research using model systems is needed to further elucidate the pathways regulated by *miR-16* and *miR-21* and to better determine the functional consequence of downregulation of *miR-16* and *miR-21* in the placenta. Additionally, more work to determine the functional effects of miRNA crosstalk – that is, miRNAs whose differential expression may have additive associations with risk for phenotype or disease – will be necessary to better understand the complex regulatory networks involved.

Poor fetal growth associated with adverse intrauterine conditions continues to be characterized. Infants classified as small for gestational age (SGA) may have experienced a number of heterogeneous environmental and placental conditions associated with describe the complex phenotype that is SGA. Because of the complexity of the conditions that may be associated with SGA status, we used SGA status as a marker suggestive of the complex milieu of conditions that may be associated with small fetal growth. Fetal malnutrition linked to growth restriction has been shown occur in a variety of conditions, including but not limited to poverty, pregnancy in women with eating disorders, and pregnancy in high altitude [28]. Maternal cigarette smoking during pregnancy is associated with an increased risk of fetal growth restriction [29], [30], and exposure to environmental toxicants *in utero*, such as those found in cigarette smoke, is associated with increased placental aberrations [31] and decreased placental function [32]. Previous work in our lab has suggested that maternal cigarette smoking during pregnancy is associated with the downregulation of *miR-16*, *miR-21*, and *miR-146a* in the placenta [9]. Thus, maternal cigarette smoking during pregnancy was included in our multivariable linear regression models because of its status as a potential confounder due to its associations with both reduced birthweight as well as reduced placental miRNA expression. A number of animal models have been generated to further study low birthweight in a controlled, experimental system [30], [33], and it will be important to consider the role of miRNA in these animal models. Because poor maternal forecasts leading to low birthweight may not accurately predict the post-birth environment and because low birthweight increases one's risk for a number of diseases later in life [2], more research is necessary to more fully understand the pathways whose dysregulation may ultimately affect birthweight.

As described described above, the multitude of factors that may contribute to altered fetal growth contribute to the relative complexity of the intrauterine environment. As concluded by Avila et al., site-to-site variability of gene expression does exist in the human placenta [34], and thus it was important for us to collect biopsies from a number of sites within each placenta sample. Since the 12 biopsies of placenta were then homogenized and combined following homogenization and prior to further analysis, we would argue that exact location within the placenta would not likely contribute to the variation in miRNA expression across the 107 placentas. A more extensive analysis of placental cell type would be important for determining differences in miRNA expression among cell types but is currently beyond the scope of our current work.

Mouillet and colleagues published important work investigating circulating levels of a set of trophoblast miRNA found in plasma and their association with fetal growth [12]. Their work was an important step in determining associations of differential miRNA expression present in plasma with fetal growth restriction. While similar in overall hypothesis, namely, that a subset of miRNA may be associated with fetal growth, our study and the work by Mouillet and colleagues differed in the sampling site, the number of samples utilized, and the candidate miRNA investigated. Our study utilized samples taken directly from term human placenta while Mouillet and colleagues used plasma samples containing circulating trophoblast miRNA. Additionally, Mouillet and coauthors' sample set contained far fewer samples than ours. In addition, we provided covariate data that Mouillet and coauthors did not. While it may indeed prove to be of important clinical utility to be able to utilize plasma samples containing circulating miRNA for future tests investigating pregnancy stage, exposure, or even fetal growth, our work was different in that we specifically were interested in characterizing miRNA expression in placentas which may be associated with aberrant fetal growth. Furthermore, we investigated miRNA expression in term placentas which may serve as a record of the complex intrauterine environment capable of programming the fetus positively or negatively. Both our work and the work of Mouillet and colleagues are important steps in further examining associations of miRNA expression with altered fetal growth.

In summary, our data suggesting that low expression of *miR-16* and *miR-21* in the placenta is associated with poor fetal growth may have many important implications. Our data are an important step in discovering miRNA expression profiles associated with low birthweight which may be powerful predictors of risk for disease later in life, such as coronary heart disease, diabetes, and hypercholesterolemia. This study is an important stepping stone in that it establishes that miRNA have the potential to predict future health outcomes based, in part, on their altered expression in the placentas of low birthweight infants. More work is needed to investigate how particular insults to the fetal environment may associate with alterations to placental miRNA expression and how these aberrant expression profiles may be associated with differential fetal growth. Future work to determine the roles of miRNA in specific pathways leading to altered fetal growth will be key to better understanding fetal growth as both a marker of the intrauterine environment as well as a developmental outcome and in better comprehending the developmental origins of health and disease.

## Materials and Methods

### Ethics statement/Placenta samples

All placenta samples used were collected as part of the Rhode Island Child Health Study (RICHS), an ongoing, population-based birth cohort at Women and Infants' Hospital in Providence, RI. The ongoing Rhode Island Child Health Study enrolls mother-infant pairs following delivery at Women and Infants Hospital in Providence, Rhode Island, USA. Term infants born small for gestational age (SGA, lowest 10^th^ percentile) based on birthweight and gestational age and calculated from the Fenton growth chart [17] were selected, and an appropriate for gestational age infant matched on infant gender, gestational age (±3 days), and maternal age (±2 years) is also enrolled. Only singleton, viable infants are included in the study. Other exclusion criteria are maternal age <18 years or a life-threatening medical complication in the mother, and congenital or chromosomal abnormality of the infant. A structured chart review was used to collect information from the maternal inpatient medical record from delivery, and mothers were subjected to an interviewer-administered structured questionnaire to obtain information on the lifestyle, demographics, and exposure histories of the participants. All study participants provided informed consent. For each placenta collected, 12 biopsies of placenta tissue, 3 from each of 4 quadrants (totaling approximately 1 g of tissue) were excised, from the maternal side of the placenta 2 cm from the umbilical cord insertion site, free of maternal decidua. The samples were placed immediately in RNAlater and stored at 4°C. At least 72 hours later, the 12 placenta biopsies were removed from RNAlater, blotted dry, snap-frozen in liquid nitrogen, homogenized using a mortar and pestle, combined to make one homogenized sample per placenta, and stored in sample tubes at −80°C until needed for examination. A structured chart review was used to collect information from the maternal inpatient medical record from delivery, and mothers were subjected to an interviewer-administered structured questionnaire to obtain information on the lifestyle, demographics, and exposure histories of the participants. All samples and information were collected under appropriate protocols approved by the Institutional Review Boards for Women and Infants' Hospital and Brown University.

### RNA extraction

RNA was extracted from placenta samples and cultured cells using the miRvana miRNA Isolation Kit (Ambion) and manufacturer protocols as described previously [20]. For tissue samples, 200 mg of homogenized tissue was used for extraction. Extracted RNA was quantified using a Nanodrop spectrophotometer and then aliquoted into single-use aliquots and stored at −80°C.

### Quantitative RT-PCR (qRT-PCR) for mature miRNA

Expression of mature miRNAs was measured using commercially available TaqMan microRNA Assays or TaqMan Gene Expression Assays (Applied Biosystems, Valencia, CA) on an Applied Biosystems 7900HT Real-Time PCR system and analyzed with 7900HT System Software. Absolute quantitation of miRNA was calculated using a standard curve generated from serial dilutions of miRNA-specific pre-miR oligonucleotides (Ambion) run on each plate for each miRNA of interest, as previously described for array analyses by Bissels and colleagues [35]. All reactions were run in triplicate on 384-well plates, and RNU-44 was used as an internal control for each sample to assess sample performance. No-RT controls were also run for each sample on each plate to assure samples were free of genomic DNA.

### Cell culture and transfection

TCL-1 placental cells were cultured as described previously [9]. Placental cells were transfected using the pre-miR miRNA precursors (Ambion) system and siPORT NeoFX transfection agenet (Ambion) following manufacturer's protocols.

### Western blot

Western blots were used to confirm that overexpression of *miR-21* resulted in decreased protein levels of PTEN compared to cells transfected with negative control. Following cell harvest and lysate collection, total protein was quantified using the BCA assay (Thermo Scientific) and manufacturer protocols. Cell lysates were then separated electrophoretically using 10% Tris-HCl gels (Bio-Rad). Proteins were transferred to Immuno-Blot PVDF membranes (Bio-Rad) through overnight night transfer at 4 degrees C and following transfer, membranes were washed with TBSt (Boston Bioproducts) and blocked with 5% milk in TBSt. For Western blot analysis, antibodies against PTEN (Invitrogen) and gamma-Tubulin (Cell Signaling) were used to visualize corresponding proteins following established protocols for staining and washing. Proteins were visualized using the Amersham ECL Western Blotting Analysis System (GE Healthcare) following the manusfacturer's protocol. Resulting images were scanned and protein quantitation was performed using Image J software.

### Statistical analysis

Birthweight percentile was calculated using the Fenton growth chart [17]. SGA was defined as the lowest 10% of birthweight percentile, as described previously by Fenton [17]. Kruskal-Wallis tests were used to determine if birthweight percentile significantly differed across quartiles of miRNA expression. Logistic regression models were used to analyze if differential expression of miRNA predicted increased risk for SGA while also considering potential confounders. All analyses were conducted in SAS 9.2 (SAS Institute, Cary, NC). Student's t-tests were used to determine if overexpression of candidate miRNA resulted in differential protein levels as determined by densitometry of PTEN and tubulin.
